# Effect of Vitamin D_3_ Supplementation on Inflammatory Markers and Glycemic Measures among Overweight or Obese Adults: A Systematic Review of Randomized Controlled Trials

**DOI:** 10.1371/journal.pone.0154215

**Published:** 2016-04-26

**Authors:** Aleksandra Zuk, Tiffany Fitzpatrick, Laura C. Rosella

**Affiliations:** 1 Dalla Lana School of Public Health, Division of Epidemiology, University of Toronto, Toronto, Ontario, Canada; 2 Public Health Ontario, Toronto, Ontario, Canada; 3 Institute for Clinical Evaluative Sciences, Toronto, Ontario, Canada; Universidad Pablo de Olavide, Centro Andaluz de Biología del Desarrollo-CSIC, SPAIN

## Abstract

**Background:**

Obesity induced low-grade chronic inflammation disrupts proper immune and metabolic function. Vitamin D deficiency increases inflammation, which is associated with cardiometabolic risk. This systematic review examines the association between oral vitamin D (VD) supplementation and circulating inflammatory biomarkers and glycemic outcomes from randomized controlled trials (RCTs) of overweight and/or obese adults.

**Methods:**

MEDLINE OVID, EMBASE and the Cochrane Central Register of Controlled Trials were searched according to a predefined protocol. Eligible RCTs included adults randomized to receive either oral VD or placebo. Two reviewers independently assessed RCTs for inclusion. Bias was assessed using the Cochrane Collaboration risk of bias tool. Mean differences were calculated comparing end-of-study sample means between the independent VD and placebo groups.

**Results:**

Eleven unique RCTs met inclusion criteria from a total of 3,383 identified citations, including 79 screened articles and 14 full text data extractions. Inflammatory and glycemic measures were reported in 7 and 10 RCTs, respectively. Most trial findings were non-significant with considerable heterogeneity in design, participants and outcomes. All but one trial was rated as either high or unclear risk of bias. Two RCTs reported significant changes in inflammatory biomarkers; however, the mean difference between groups was not statistically significant: C-reactive protein 0.19 mg/L (p = 0.88); Tumor Necrosis Factor -0.54 pg/ml (p = 0.20). Two other trials found significant mean differences in fasting plasma glucose -0.32 mmol/L (p = 0.03), Hemoglobin A1c -0.13% (p = 0.04), and Homeostatic Model Assessment -0.86 (p = 0.02) following VD supplementation.

**Conclusions:**

Overall, there is no clear established benefit of VD supplementation on inflammatory biomarkers among overweight/obese adults. Baseline serum VD possibly influences the effect of VD repletion on inflammatory markers. Risk of bias was present in most studies, thus supporting the need for higher quality studies in this area to more conclusively understand the role VD supplementation has on inflammatory pathways.

## Introduction

Obesity is a growing public health problem [1], associated with increased morbidity and mortality risks [2], and contributes to the burden of chronic disease [3–5]. In the past decade, there has been evidence to suggest that obesity induces low-grade chronic inflammation, which disrupts the proper functioning of the immune and metabolic state [6, 7]. Obesity-associated inflammatory response impairs the immune function, signals additional inflammatory pathways and contributes to obesity-related health conditions [7–9]. In a suitable environment with appropriate stimuli, obesity promotes a pro-inflammatory state by increasing circulating levels of inflammatory cytokines [10]. Interleukin 6 (IL-6), Tumour Necrosis Factor (TNF)-α are shown to be positively correlated with increased adiposity [11] and C-reactive protein (CRP) increases with visceral obesity [12]. Furthermore, obesity decreases adiponectin levels, which have an important role of inhibiting the inflammatory process [13–15] and increasing the expression of anti-inflammatory cytokine, interleukin (IL-10) [16, 17].

Several studies have reported that vitamin D may reduce the inflammatory response and improve insulin sensitivity [18]. It is no surprise then that vitamin D deficiency has been observed in, and suggested to contribute to various obesity-related conditions such as insulin resistance [19], diabetes [20, 21] and cardiovascular disease [22]. Moreover, markers of oxidative stress and inflammation are shown to be elevated in persons with low serum vitamin D 25(OH)D concentrations, however, results are not always consistent [23–26]. Vitamin D inadequacy is prevalent in many countries [27]. There are several factors that contribute to suboptimal levels of vitamin D including melanin pigment, seasonality, aging, and geography [28]. As such, the role that vitamin D may have on obesity through these inflammatory markers could provide insight into the benefit of supplementation given the global rise in obesity [29]. Therefore, the primary aim of this review is to examine the association between oral vitamin D supplementation and circulating inflammatory biomarkers based on results from randomized controlled trials among overweight and/or obese adults. The secondary aim was to assess the effect of vitamin D supplementation on glucose and insulin sensitivity measures in the same population.

## Methods

A pre-defined review protocol was developed and underwent third-party adjudication (SS, AT) prior to initiating the review. The search strategy for this review was devised with the assistance of a professional librarian (LP). The protocol has not published at this time. However, this systematic review complies with the preferred reporting items of PRISMA for systematic reviews [30].

### Criteria for considering studies for this review

#### Types of studies

We conducted a systematic review of randomized controlled trials (RCTs), investigating the effect of vitamin D supplementation on an *a priori* list of outcomes. We excluded single arm trials, commentaries, editorials, cross-sectional, retrospective, case-cross over and pilot studies, as well as trials where randomization was broken or not reported.

#### Types of participants

We included adult participants 18 years of age or older defined as being overweight or obese, i.e., body mass index (BMI) ≥ 25 kg/m^2^ (overweight), BMI ≥ 30 kg/m^2^ (obese), or waist circumference (WC) ≥ 88 cm for women, and WC ≥ 102 cm for men [31]. As well, we included lower ethnic-specific BMI cut-off points as recommended by the World Health Organization to support increased risk (BMI ≥ 23 to 27.5 kg/m^2^) and high risk (BMI ≥ 27.5 kg/m^2^) among Asian populations [32]. Trials enrolling participants with chronic kidney disease, hemodialysis, renal disease or kidney transplantation were excluded because of the associated severe vitamin D deficiency risk and the potential treatment requirement with vitamin D sterols [33]. No other restrictions were applied to studies in this review.

#### Types of interventions

Trials administering vitamin D orally in any dose, duration and form (e.g. cholecalciferol (D_3_), ergocalciferol (D_2_), or alfacalcidol), including those with or without a calcium co-intervention, were eligible for inclusion. Studies administering vitamin D in the form of food (i.e., fortified yogurt drink) or by intramuscular injection were excluded due to the distinct drug absorption and bioavailability differences between the two administration routes [34, 35]. No other restrictions on baseline serum vitamin D levels, dosage, or trial duration were applied. Trials comparing the effects of oral calcium (primary intervention) plus vitamin D were also eligible for inclusion if there was a clear and measurable difference between the comparison groups. For instance, calcium supplement plus vitamin D compared to placebo, or calcium plus vitamin D compared to a lower supplemental dose of vitamin D. Control group parallel to the intervention group was defined as either an orally administered supplement with no active form of vitamin D; a substantially lower vitamin D dose; or no concurrent oral therapy.

#### Types of outcome measures

The following end-of-study (EOS) inflammatory markers, insulin sensitivity and glycemic measures were reviewed: acute phase: C-reactive protein (CRP) or high-sensitivity (hs-CRP); pro-inflammatory cytokines: Tumor Necrosis Factor (TNF)-α and Interleukin 6 (IL-6); anti-inflammatory cytokines: Interleukin 10 (IL-10) and adipocytokines: leptin and adiponectin; glucose measures: Fasting Plasma Glucose (FPG), and Hemoglobin A1c (HbA1c); insulin sensitivity: Fasting insulin levels (FIL), euglycemic-hyperinsulinemic clamp (EHC), homeostatic model assessment (HOMA-IR) and quantitative insulin sensitivity check index (QUICKI).

### Search methods for identification of studies

The Cochrane Database of Systematic Reviews (CDSR) and the Database of Abstracts of Reviews of Effects were searched for preexisting reviews. No systematic reviews focusing on vitamin D supplementation and inflammation among overweight or obese adults were found. The electronic bibliographic databases of MEDLINE OVID, EMBASE and Cochrane Central Register of Controlled Trials (CENTRAL) were searched from date of inception to week 41, 2013. No language limits were applied. The full search strategy, with medical subject headings (MeSH), keywords and limits, are presented in the supplementary files (S1 File).

### Data collection

#### Study selection

After duplicate titles were removed, two reviewers (AZ, TF) independently screened titles and abstracts according to our predefined criteria. Next, both reviewers independently assessed the inclusion eligibly of all full-text articles for abstracts that passed the initial screening stage; a third independent reviewer (LR) reconciled any disagreements. Kappa statistics [36] was used to evaluate inter-rater reliability of the title and abstract screen between reviewers (AZ, TF) prior to resolving any disagreement differences. The observed agreement was 98% between the two reviewers with Kappa = 0.61 (95% CI: 0.50, 0.71). According to Landis and Koch [37], the strength of agreement is considered ‘substantial’ [38].

#### Data abstraction and quality assessment of trials

Data abstraction was conducted independently by two reviewers (AZ, TF) using an *a priori* developed abstraction form, which was piloted on a small number of selected studies. Reviewers assessed the methodological quality of selected trials using the Cochrane Collaboration Risk of Bias Tool (CCRBT) as described in the Cochrane Handbook for Systematic Reviews of Interventions [39]. Assessment of risk was rated as ‘high’ (-), ‘low’ (+) or ‘unclear’ (?) across the following six domains: 1) sequence generation (selection bias); 2) allocation concealment (selection bias); 3) blinding of participants, personnel (performance bias) and outcome assessors (detection bias); 4) incomplete outcome data; 5) selective outcome reporting; and 6) other sources of bias.

### Statistical Analysis

Twelve continuous measures (CRP, TNF-α, IL-6, IL-10, adiponectin leptin, FPG, HbA1c, HOMA-IR, QUICKI, FIL and EHC) were summarized. All measures are reported in SI units; any measurement reported in non-SI units was converted using the appropriate conversion factors. Data extraction of all outcome measures, including both within and between group end-of-study differences, for the vitamin D treatment and control groups. Where not already provided, end-of-study mean differences (Δ) were calculated based on the data presented in the trials to compare end-of-study sample means between the two independent vitamin D and placebo group [40]. As well, ranges, standard deviations and standard errors were also extracted where available. For consistency, all variance measures have been reported in standard error of the mean difference (±SEM), where possible. Trials reporting median values were assumed to be normally distributed. Due to large variances in study design, interventions, and participants outcome data was not pooled across trials. All calculations were performed using Review Manager software (RevMan) [Computer program]. Version 5.3; Copenhagen: The Nordic Cochrane Centre, The Cochrane Collaboration, 2014).

## Results

### Description of studies

#### Search results

**Fig 1:**
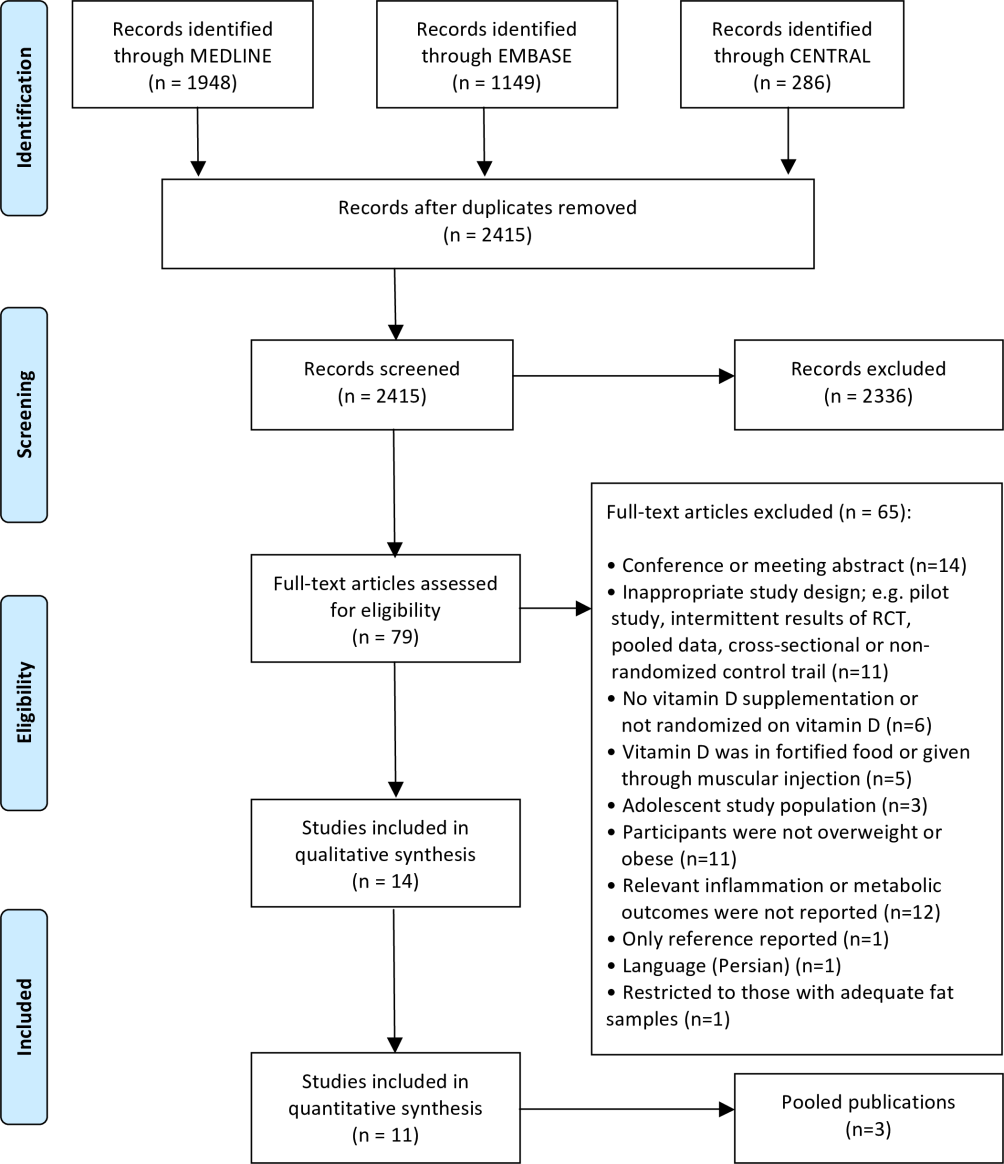
Study selection flow diagram. PRISMA flow diagram of search results following study section procedure assessing vitamin D supplementation and circulating inflammatory markers, glucose, and insulin sensitivity markers among randomized controlled trials (RCTs) of overweight and/or obese adults. PRISMA, Preferred Reporting Items for Systematic Reviews and Meta-Analyses [30].

A total of 3,383 citations were identified, including 79 articles screened at full-text level and 14 that underwent full data extraction. The Preferred Reporting Items for Systematic Reviews and Meta-Analysis (PRISMA) flow diagram [30] is presented in (Fig 1). Three trials, Beilfuss et al. 2012 [41], Jorde et al. 2010a [42] and Jorde et al. 2010b [43], referenced the same study protocol and population. Duplicate data was reported for certain outcomes (e.g. hs-CRP, HOMA-IR, QUICKI); e.g., Beilfuss et al [41] combined two vitamin D intervention groups into one vitamin D group for inflammatory marker (hs-CRP) and insulin sensitivity measures (QUICKI, HOMA-IR). Therefore, the results of the three trials are reported separately, but the risk of bias and quality assessment is documented jointly [41–43]. Similarly, two other trials, Carrillo el al. 2012 [44] and Carrillo et al. 2013 [45], also referenced the same study population but with no overlapping outcomes [44, 45]. As such, the final analysis consisted of 11 unique trial protocols identified from 14 publications [41–54].

#### Description of included studies and participants

Study design and baseline participant characteristics are presented in Tables 1 and 2. Over half of the trials were conducted in the United States [44–46, 50–52, 54]; three in Europe [41–43, 47, 48]; and two in Asia [49, 53]. All but two of the studies were parallel-group RCTs consisting of oral vitamin D_3_, with or without calcium, along a placebo-controlled supplement, with or without calcium. The remaining two trials were multiple-arm randomized controlled trials with three and four-arm designs [41–43, 52]. A total of 1698 subjects were randomized, with 1324 subjects remaining at the end of study. Most trials enrolled middle-age populations with the exception of three trials: two that exclusively enrolled young adults less than 35 years and one enrolling adults older than 65 years of age. Over half (795/1324) of the participants were female; however, one trial exclusively enrolled men [49]. Three trials selected participants based on central adiposity [49, 50, 54]; the remaining used ethnic-specific or standard BMI cut-offs for overweight and obesity. One trial did not explicitly report selecting participants on the basis of weight, but the mean BMI of participants at baseline was 26.7 kg/m^2^ with tight confidence intervals, suggesting the overwhelming majority of participants were overweight or obese [46]. All baseline values of reported outcomes are presented in Table 3.

**Table 1:** Randomized controlled trial study characteristics.

Author, Year (ref) Country	Eligibility Age (years)	No. arms	Treatment groups †	Average Dose VD (IU)	FOA ⱡ; Duration	CO (%)ⱡ	Co-morbidities ⱡ	SSR ⱡ	RFF ⱡ	ITT ⱡ	Comments ⱡ
Maki, 2011 [54] USA	18–79	2	I = MVMD; P = MVM	1,200	Daily; 8 weeks	I = 98.5; P = 96	1. Centrally obese (waist circumference (wc) ≥ 102 cm or ≥ 88 cm for men/women);	Yes	Yes	Mod.	1. Enrollment mid-summer to fall.
Carrillo, 2012 [44]; Carrillo, 2013 [45] USA	18–35	2	I = VD; P = PL; C = CA+SS+NS+PA	4,000	Daily; 12 weeks	I = 93; P = 93	1. Overweight or obese (BMI: 25–40kg/m²); 2. Activity and VO_2_ max low or very low	NR	Yes	No	1. Enrollment October to March; 2.Matched for age and weight
Nagpal, 2009 [49] India	≥ 35	2	I = VDc; P = PLc (sachets)	120,000 ^1^	Biweekly; 6 weeks	NR ^2^	1. Centrally obese (wc) ≥ 78 cm)	Yes	No	Yes	1. Enrollment Aug 2006 to Mar 2007.
Pittas, 2007 [46] USA	≥ 65	2	I = VD + CA; P = PL	700 ^3^	Daily; 3 years	I = 93; P = 93	1. Overweight or obese;	Yes	Yes	No	1. Post hoc analysis; 2. VD tablet dose miscalculated in OT; 3.Enrollment Oct 1992 to Feb 1996; 4. Stratified by NFG (< 5.6 mmol/L) and IFG (5.6 to 6.9 mmol/L).
Wamberg, 2013 [47] Denmark	18–50	2	I = VD; P = PL	7,000	Daily; 26 weeks	I = 95; P = 94	1. Obese (BMI> 30kg/m²); 2. Plasma 25(OH)D < 50 nmol/L	Yes	No	No	1. Enrollment Jan 2010—Jul 2012; 2.Randomization stratified by gender and by fasting glucose (6.1 mmol/L)
Zittermann, 2009 [48] Germany	18–70	2	I = VDo; P = PLo; C = WRP	3,332	Daily; 1 yr	NR ^4^	1. Overweight or obese (BMI> 27 kg/m²)	Yes	Yes	No	1. Enrollment Dec to Nov
Beilfuss, 2012 [41]; Jorde, 2010a [42]; Jorde, 2010b [43] Norway	21–70	3	I = DD+DP; P = PP; C = CA+HD/PA	DD = 40,000; DP = 20,000 ^5^	Weekly; 1 yr	DD = 95;DP = 96;PP = 96	1. Overweight or obese (BMI: 28–47 kg/m²)	Yes	DD: NR ^6^; DP: Yes; PP: NR ^6^	No	1. Enrollment Nov 2005—Oct 2006; 2. Stratified by gender and smoking status; 3. All three publications are based on data from one trial protocol
Davidson, 2013 [50] USA	≥ 40	2	I = VDs; P = PLs	NR ^7^	Weekly; 1 yr	I/P = 100 ^8^	1. Vitamin D (≤ 75 nmol/L); 2.Centrally obese (wc ≥40 or ≥35 inches for men /women); 3.Prediabetes (A1C: 5.8 to 6.9%), OGTT (FPG 110–125 mg/dL or 2hr glucose 140–199 mg/dL)	NR	No	Mod.	1. Enrollment Mar 2009—Jun 2012; 2. Guidelines for defining diabetes with confirmed A1C levels changed as per ADA.
Harris, 2012 [51] USA	≥ 40	2	I = VD; P = PL; C = CA (600 mg/d)	4,000	Daily; 12 weeks	I = 94.4; P = 96.5	1. Overweight or obese (BMI: 25–40 kg/m²); 2.Prediabetes (FPG ≥100 mg/dL or A1C: 5.8 to < 7%)	Yes	Yes	No	1. Enrollment Jul 2008—Feb 2011; 2. Guidelines for defining diabetes changed as per ADA. Trial originally designed to include pre-diabetes and exclude diabetes; 3. Randomized by age and sex strata.
Mitri, 2011 [52] USA	≥ 40	4	I = VDCA+VDCP; P = VPCA+VPCP; C = CA (800 mg/d)	2,000	Daily; 16 weeks	I = 89; P = 85	1. Overweight or obese (BMI ≥25 kg/m² or ≥23 kg/m² if Asian); 2. Pre-diabetes (FPG ≥ 100 mg/dL or 2hr glucose ≥140 mg/dL or A1C ≥ 5.8%)	NR	Yes	Yes	1. Enrollment Oct 2007—Jul 2009; 2.Adjusted for season of study entry (Jan-Mar vs. Apr-Jun; Jul-Sep vs. Oct-Dec); 3.Permutated blocks, stratified by age and BMI
Zhu, 2013 [53] China	18–25	2	I = CA (600 mg/d) + VD; P = None; C = CR	125	Daily; 12 weeks	I = 95.8	1. Overweight (BMI 24–27.9 kg/m²) or obese (BMI > 28 kg/m²);	No	Yes	No	1. Enrollment Apr—Dec 2011

**† C** = Co-intervention; **CA** = Calcium tablet (500 mg/day unless otherwise noted); **CR** = Calorie restriction (500 kcal/day); **DD** = 2 vitamin D tablets; **DP** = 1 vitamin D tablet and 1 placebo tablet; **HD/PA** = Written and oral information and recommendations on healthy diet and physical activity; **I** = Intervention arm; **MVM** = Multivitamin and mineral; **MVMD** = Multivitamin and mineral with Vitamin D; **No.** = Number; **NS** = Nutritional Shake (360 kcal, 8 g fat, 54 g carbohydrate, 20 g milk protein isolate, 100 IU vitamin D and 300 mg calcium); **P** = Placebo arm; **PA** = Physical activity (progressive resistance training); **PL** = Placebo; **PLc** = Placebo sachets; **PLo** = Placebo Migliol oil; **PLs** = Placebo solution via oral syringes; **PP** = 2 placebo tablets; **SS** = Sunscreen; **VD** = Vitamin D tablet; **VDc** = Vitamin D sachets; **VDCA** = Vitamin D plus calcium; **VDCP** = Vitamin D plus calcium placebo; **VDo** = Vitamin D3 Vigantol oil; **VDs** = Vitamin D dissolved in median chain triglyceride via oral syringes; **VPCA** = Vitamin D placebo plus calcium; **VPCP** = Vitamin D placebo plus calcium placebo; **WRP** = Weight reduction program; **25(OH)D** = circulating vitamin D metabolite.

**ⱡ CO** = Compliance; **FOA** = Frequency of administration; **ITT** = Intention to treat analysis; **Mod** = Modified; **RFF** = Recorded Food Frequency; **SSR** = Smoking Status Reported; **ADA:** American Diabetes Association; **BMI** = Body mass index; **FPG** = Fasting plasma glucose (mg/dL); **A1C** = Hemoglobin A1c (%)**; IFG** = Impaired fasting glucose (fasting plasma glucose 5.6 to 6.9 mmol/L); **NFG** = Normal fasting glucose (fasting plasma glucose < 5.6 mmol/L); **OGTT** = Oral glucose tolerance test; **VO2 max** = Maximal oxygen consumption; **WC** = Waist circumference.

1) Vitamin D dose given fortnightly x3

2) NR: those who accepted the second VD or PL dose also accepted the third

3) Authors report dose inconsistencies during the 3-year study design

4) NR: compliance was assessed by measuring vitamin D 25(OH)D concentrations

5) Vitamin D group, 2 tablets (DD) = 40,000 IU & Vitamin Placebo group (DP) = 20,000 IU given weekly

6) NR: original trial documents food frequency

7): vitamin D mean weekly dose 88,865 16,154 IU (range 64,731–134,446), based on supplementation formula (100—baseline serum (25-OHD) x kg body weight x 15 IU / week)

8) authors report 100% compliance.

**Table 2:** Randomized controlled trial subject characteristics.

Author, Year (ref)	No.S; No.R	T.No. (EOS); LTFU	No. per arm (EOS)	Female n (%)	Age (SD)	Ethnicity § n(%)	Mean BMI (SD)	Mean (SD) serum 25(OH)D (nmol/L) at baseline
Maki, 2011 [54]	80; 60	59; 1.7%	I = 30; P = 29	I = 23 (74.2); P = 22 (75.9)	I: 50.3 (2.5); P: 54.3 (2.0)	I: 30 (96.8) W; P: 27 (93.1) W	I: 31.7 (1.1); P: 31.7 (1.0)	I: 64.4 (20.6); P: 67.9 (19.9)
Carrillo, 2012 [44]; Carrillo,2013 [45]	NR; 34	23; 32.4%	I = 10; P = 13	I = 5 (50.0); P = 7 (53.9)	I: 26.2 (5.1); P: 26.0 (4.5)	NR	I: 30.6 (3.1); P: 31.9 (3.3)	I: 51.9 (20.7); P: 45.2 (16.2)
Nagpal, 2009 [49]	NR; 100	65; 35.0%	I = 32; P = 33	I = 0; P = 0	I: 42.4 (6.6); P: 45.0 (9.2)	Indian only	I: 26.7 (4.5); P: 26.0 (3.5)	I: 36.5 (14.5); P: 30.0 (12.5)
					**NFG**		**NFG**	**NFG**
Pittas, 2007 [46]	848; 445	314; 28.5%	I = 153; P = 161	I = 87 (56.9); P = 94 (58.4)	I: 70.6 (0.4); P: 71.7 (0.4)	White only	I: 26.1 (0.3); P: 26.2 (0.3)	I: 81.4 (38.5); P: 70.6 (26.9)
					**IFG**		**IFG**	**IFG**
					I: 71.1 (0.7); P: 71.3 (0.8)		I: 27.8 (0.6); P: 28.1 (0.7)	I: 71.2 (36.8); P: 81.2 (32.4)
Wamberg, 2013 [47]	88; 52	43; 17.3%	I = 22; P = 21	I = 18 (69.2); P = 19 (73.1)	I: 39.5 (8.0); P: 41.2 (6.8)	Danish	I: 36.1 (3.4); P: 35.0 (3.2)	I: 34.5 (10.8); P: 34.6 (10.3)
Zitterman, 2009 [48]	298; 200	165; 17.5%	I = 82; P = 83	I = 51 (62.2); P = 60 (72.3)	I: 47.4 (10.3); P: 48.8 (10.1)	Germans	I: 33.7 (4.1); P: 33.0 (4.3)	I: 30.0 (17.5); P: 30.3 (20.1)
Beilfuss, 2012 [41] §§	626; 445	332; 25.4%	I = 220; P = 112	204 (61.4)¶	50 (23–70) ¶ *	Norwegians	I: 33.5 (28.7–46.1); P: 34.7 (28.6–47.1) *	I: 54.3 (15.4–111.5); P: 52.4 (18.5–99.4) *
					DD: 46.3(11.3);		DD: 34.8 (4.0);	DD: 58.7 (21.2);
Jorde, 2010a [42] §§	626; 438	330; 24.7%	DD = 11; DP = 104; PP = 149	DD = 94(62.7);DP = 89(64.0);PP = 98(65.8)	DP: 47.3(11.9);	Norwegians	DP: 34.4 (3.8);	DP: 56.7 (21.2);
					PP: 48.9(11.0)		PP: 35.1 (3.8)	PP: 58.8 (21.0)
Jorde, 2010b [43] §§	626; 445	307; 31.0%	DD = 10; DP = 98; PP = 105	DD = 63(60.6); DP = 62(63.2);PP = 68(64.8)	NR	Norwegians	NR	NR
Davidson, 2013 [50]	155; 117	99; 15.4%	I = 56‡; P = 53‡	I = 36 (64.0); P = 38 (71.0)	I: 52.3 (8.0); P: 52.5 (7.0)	I: 51 (91.0) L; P: 44 (83.0) L	I: 32.1 (4.7); P: 32.9 (4.3)	I: 54.9 (11.2); P: 54.9 (12.0)
Harris, 2012 [51]	989; 100	89; 11.0%	I = 43; P = 46	I = 19 (43.5); P = 26 (56.5)	I: 57.0 (10.4); P: 56.3 (12.3)	African American only	I: 32.6 (4.1); P: 31.9 (4.0)	I: 39.6 (12.9); P: 38.2 (15.5)
Mitri, 2011 [52]	911; 92	88; 4.3%	I = 46; P = 46	I = 24 (52.2); P = 23 (50.0)	I: 57.0 (13.5); P: 58.0 (13.5)	I: 37 (80.4) W; P: 35 (76.1) W	I: 32.5 (6.8); P: 32.0 (6.8)	I: 60.9 (18.6); P: 61.4 (18.6)
Zhu, 2013 [53]	129; 53	43; 18.9%	I = 22; P = 21	I = 21 (95.5); P = 18 (85.7)	I: 20.1 (1.1); P: 20.3 (0.8)	Chinese only	I: 26.0 (1.8); P: 25.7 (1.7)	NR

**EOS** = end of study; **I** = Intervention arm; **P** = Placebo arm; **LTFU** = lost to follow-up; **No.** = Number; **NR** = Not Reported; **No.S =** Number Screened; **No.R =** Number Randomized; **T.No.** = Total Number; **NFG** = normal fasting glucose (fasting plasma glucose < 5.6 mmol/L)**; IFG** = impaired fasting glucose (fasting plasma glucose 5.6 to 6.9 mmol/L)**; DD** = two vitamin D tablets; **DP** = 1 vitamin D tablet + 1 placebo tablet; **PP** = two placebo tablets

**§** Nationality reported when ethnicity not provided; **W** = white; **L** = Latino

**¶** treatment specific estimated not available

* Median & range provided (mean not reported)

‡ completed 3-months (n = 109)

**§§** Reference the same study protocol and population.

**Table 3:** Randomized controlled trial subject characteristics baseline measures.

	Glucose Measures		Insulin Sensitivity			Inflammatory Markers				Adipokines	
	Mean (SD)		Mean (SD)			Mean (SD)				Mean (SD)	
Study	FPG (mmol/L)	HbA1c (%)	HOMA-IR	QUICKI	FIL (pmol/L)	(hs-)CRP (mg/L)	TNF-α (pg/ml)	IL-6 (pg/ml)	IL-10 (pg/ml)	Adiponectin (mg/ml)	Leptin (ng/ml)
**Outcome: Inflammatory and glycemic markers**											
**Carrillo, 2012; Carrillo, 2013**	I: 5.4 (0.5); P: 5.3 (0.4)	-	I: 3.8 (2.3);P: 3.6 (2.4)	-	I: 108.5(62.3); P: 105.8(71.2)	I: 2.8(2.7); P: 3.8(2.8)	I: 2.3 (1.1); P: 1.9 (1.3)	I: 1.6(0.7); P: 1.1(0.6)	-	-	-
**Nagpal, 2009**	-	-	I: 1.5 (1.2); P: 1.3 (0.9)	I: 0.2 (0.02); P: 0.2 (0.02)	-	I / P: < 0.3*	-	-	-	-	-
	**NFG**		**NFG**			**NFG**		**NFG**			
**Pittas, 2007** †	I: 5.1 (0.3); P: 5.1 (0.3)	-	I: 1.3 (1.0); P: 1.1 (1.1)	-	**-**	I: 2.9 (3.1); P: 2.9 (3.2)	-	I: 3.4 (4.2); P: 3.9 (4.3)	**-**	-	-
	**IFG**		**IFG**			**IFG**		**IFG**			
	I: 6.0 (0.3); P: 6.0 (0.3)		I: 2.2 (1.3); P: 2.3 (0.7)	-	-	I: 2.5 (2.0); P: 2.4 (2.8)	-	I: 4.3 (5.4); P: 2.9 (2.8)	-	-	-
**Wamberg, 2013**	I: 5.3 (0.4); P: 5.5 (0.5)	-	I: 3.1 (NR) ⱡ; P: 3.2 (NR)	-	I: 72.9 (NR) ⱡ; P: 82.2 (NR)	I: 7.4 (NR) ⱡ; P: 5.4 (NR)	-	I: 2.3 (NR) ⱡ; P: 2.5 (NR)	-	I: 5.9 (NR) ⱡ; P: 5.1 (NR)	I: 34.4(16.8); P: 33.3(17.1)
**Zittermann, 2009**	I: 5.7 (0.8); P: 5.6 (1.2)	I: 5.6 (0.4); P: 5.7(0.6)	-	-	-	I: 0.4 (0.3); P: 0.4 (0.6)	I: 7.8 (3.2); P: 8.1 (3.4)	I: 8.9(15.2); P: 7.8(12.3)	-	-	-
	DD: 5.3(0.6);	DD: 5.6 (0.3)	DD: 4.7 (2.9);	DD: 0.3 (0.02);	DD: 119 (65);	-	-	-	-	-	-
**Jorde, 2010a** †† §§	DP: 5.3 (0.6);	DP: 5.6 (0.4)	DP: 4.3 (2.2);	DP: 0.3 (0.02);	DP: 108 (50);	-	-	-	-	-	-
	PP: 5.3 (0.5)	PP: 5.7 (0.3)	PP: 4.9 (2.9)	PP: 0.3 (0.02)	PP: 121 (65)	-	-	-	-	-	-
**Beilfuss, 2012** ** §§	-	-	I: 3.7 (NR) ⱡ; P: 4.1 (NR)	I: 0.3 (NR) ⱡ; P: 0.3 (NR)	-	I: 2.4 (NR) ⱡ; P: 2.5 (NR)	I: 1.5(NR) ⱡ; P: 1.5(NR)	I: 1.1 (NR) ⱡ; P: 1.2 (NR)	-	-	-
**Outcome: Inflammatory markers only**											
**Jorde, 2010b** †† §§	-	-	-	-	-	NR	-	-	NR	-	-
**Maki, 2011**	-	-	-	-	-	I: 4.0 (1.2); P: 3.3 (0.9)	-	-	-	-	-
**Outcome: Glycemic markers only**											
**Davidson, 2013**	I: 5.5 (0.5); P: 5.4 (0.5)	I: 6.1 (0.3); P: 6.1 (0.4)	I: 2.1 (NR); P: 2.2 (NR)	-	-	-	-	-	-	-	-
**Harris, 2012**	I: 5.2 (0.4); P: 5.5 (1.0)	I: 6.1 (0.4); P: 6.1 (0.5)	I: 4.0 (2.6); P: 3.5 (1.5)	-	I: 90.2 (38.8); P: 97.0 (54.3)	-	-	-	-	-	-
**Mitri, 2011** ⱡ ⱡ	I: 5.1 (0.5); P: 5.2 (0.5)	I: 5.9 (0.3); P: 5.9 (0.4)	-	-	-	-	-	-	-	-	-
**Zhu, 2013**	I: 4.6 (0.3); P: 4.6 (0.3)	-	-	-	I: 72.9 (35.0); P: 63.0 (27.9)	-	-	-	-	-	-

**NR** = Not Reported; **I** = Intervention arm; **P** = Placebo arm; **DD** = (2 Vitamin D tablets), **DP** (1 vitamin D tablet and 1 placebo tablet), and **PP** (2 placebo tablets).; **SD** = Standard Deviation

**†** Post hoc analysis stratified participants with normal fasting glucose **(NFG),** fasting plasma glucose < 5.6 mmol/L, and impaired fasting glucose **(IFG),** fasting plasma glucose 5.6 to 6.9 mmol/L;

**ⱡ** Means not reported, median values and ranges provided

**††** Three arms in this study

**Only the results for DD + DP vs PP are shown

**ⱡ ⱡ** Trial was 2x2 factorial design for calcium and vitamin D supplementation. Only those results related to vitamin D are shown

**§§** Reference the same study protocol and population

* Mean hs-CRP not reported in the Intervention (I) or the Placebo (P) arm, only categorical distribution: <0.3 mg/L, 0.3–1.0 mg/L, and >1.0 mg/L. Over 80% of participants had baseline hs-CRP levels <0.3 mg/l and only 1 participant in each arm had hs-CRP values greater than 1.0 mg/l at baseline.

Varying guidelines exist for defining optimal serum concentrations of 25-hydroxyvitamin-D 25(OH)D in humans. The Institute of Medicine, Food and Nutrition Board define vitamin D deficiency when serum 25(OH)D levels are below 30 nmol/L, inadequacy if 25(OH)D is between 30 and 50 nmol/L, and sufficiency when 25(OH)D is greater than 50 nmol/L [55]. According to baseline mean serum 25(OH)D levels, RCTs were categorized as involving inadequate vitamin D levels in five of the trials. Notably, one trial did not report baseline 25(OH)D concentrations [53]. The Endocrinology Society [56] defines vitamin D deficiency as a 25(OH)D levels below 50 nmol/L and insufficiency as a 25(OH)D level between 52.5 nmol/L and 72.5 nmol/L). Therefore, five trials would be considered deficient and four insufficient according to baseline mean serum 25(OH)D levels. Among trials reporting end-of-study serum vitamin D levels, seven of ten trials had mean serum 25(OH)D concentrations greater than 80 nmol/L in the treatment arm [41–48, 50, 51].

#### Description of included interventions

All trials provided D_3_ supplementation. Most trails supplied vitamin D in capsule form; however, two trials supplied vitamin D in oil form [48, 50] and one provided vitamin D sachets [49]. Dosage and duration of supplementation varied. Among trials reporting daily vitamin D_3_ supplementation (n = 8), the average dose per day ranged from 7,00 IU to 7,000 IU [44–48, 51–54]. In the remaining trials, vitamin D supplementation was provided as follows: 1) 120,000 IU fortnightly [49]; 2) weekly doses of 20,000 or 40,000 IU [41–43]; and 3) weekly pre-filled oral syringes that were based on an individualized supplementation formula from a pharmacokinetic study [50]. Co-interventions occurred in over half (8 of 11) of the trials, mostly with calcium supplementation [41–46, 51–53]. Other co-interventions included sunscreen, nutritional shakes, weight resistance training [44, 45], multi-vitamin use [54], healthy diet and physical activity [41–43], weekly nutrition education with dietary counseling [48] and calorie restriction [53].

#### Quality of included studies

**Fig 2:**
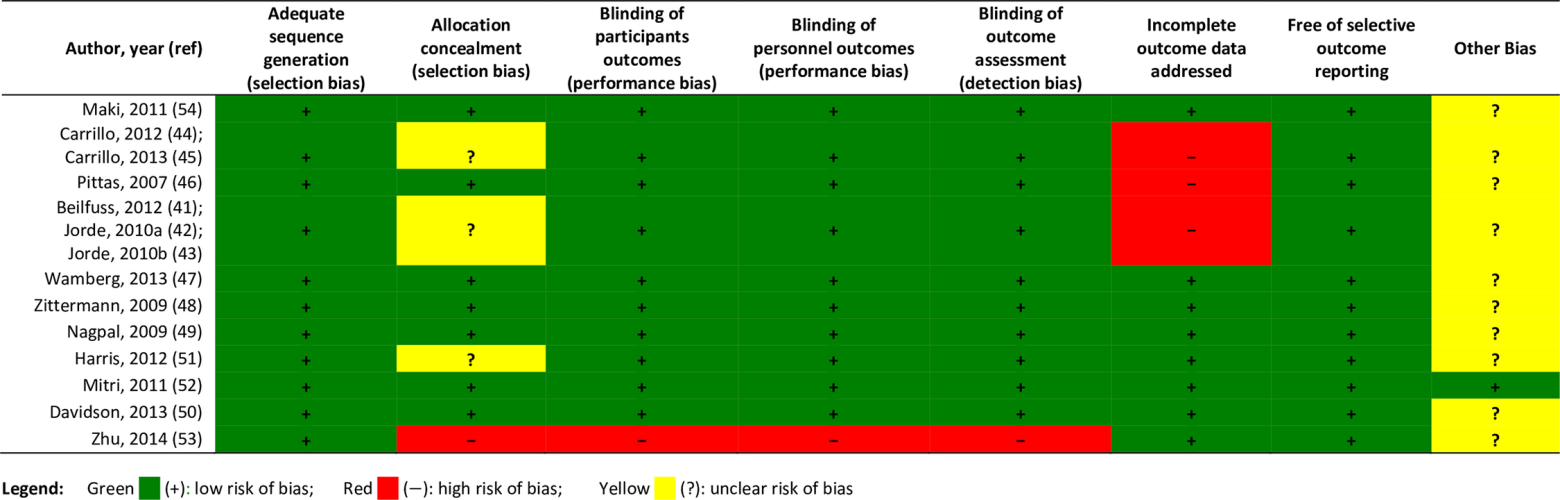
Quality assessment using Cochrane Collaboration Risk of Bias Tool. Green (+) indicates low risk of bias; Red (-) indicates high risk of bias; and Yellow (?) indicates unclear risk of bias.

Quality assessment identified at least one risk of bias present in almost all trials; overall the trials had a high or unclear risk of bias (Fig 2). Insufficient description of allocation concealment led to unclear selection bias ratings in three of the eleven distinct protocols. Zhu et al. [53], an open-label trial, was identified as having high risk for selection bias, performance bias and detection bias. The remaining seven trials reported the minimum methodological criteria for allocation concealment [57]. Bias due to attrition was a concern in three trials, with missing outcome data ranging from 24% to 35% [41–46].

Reporting bias [58] and lost-follow-up [59, 60], impacted the methodological quality in Pittas et al. [46], a *post hoc* analysis of vitamin D on blood glucose and inflammatory markers from an RCT conducted 10-years earlier with primary skeletal outcomes. Notably, participants who had withdrawn from the original trial significantly differed from those that did not with respect to smoking status (10% versus 4%, respectively; p-value = 0.02) [61]. Further, the authors did not address the fact that mid-way through the original trial product manufactory inconsistencies with vitamin D tablets were identified [61]. As well, incompleteness of stored specimen samples was a potential source of bias [46].

Attrition bias was also a concern in Jorde et al. 2010a [42], as there were significant differences between those who completed the trial compared with those who dropped out (e.g. lower serum 25(OH)D, higher BMI, more smokers and younger age). The two other trials using these data failed to include the aforementioned attrition differences [41, 43]. Differential dropout rates between study treatments were also present in two trials [47, 53]; Wamberg et al. [47] and Zhu et al. [53] report a 15% lost to follow-up in the vitamin D group and 20% and 23%, respectively in the control group. Intention-to-treat (ITT) analyses were conducted in four studies [49, 50, 52, 54]; two of which used modified-ITT with included 1.7% to 35% lost to follow-up rates [50, 54]. Other sources of bias include failing to disclose prior ethical approval [54], lacking a ClinicalTrials.gov registry number (NCT) [49, 54], and lacking clarity involving the role of industry following supplementation, support or funding [41–45, 48, 54]. Further bias stemmed from inconsistent reporting; e.g. Harris el al. [51] identified their trial as a “randomized, placebo-controlled, parallel group trial”; however, the trial protocol described the RCT as a “double-blind”, masking subject, caregiver, investigator and outcome assessor. Serious potential for bias were noted in some trials failing to provide similar co-interventions; e.g., because of budget constraints, Zhu et al. [53] did not provide a matching placebo pill. Similarly, Davidson et al. [50] encouraged subjects not to change current vitamin/mineral use, and did not provide any additional information about concurrent supplement use. Furthermore, this trial did not display baseline randomized subject characteristics or end-of-study participant characteristics; glucose and insulin sensitivity indices are reported only at 3-months using modified-ITT. Finally, only two trials adequately compared intervention groups with total randomized participants [42, 52], rather than solely documenting statistical differences in baseline characteristics between groups [57].

#### Markers of inflammation

Seven trial protocols examined changes in inflammatory markers (Table 4); only one of the trials did not additionally report either glucose or insulin sensitivity measures [54]. Beilfuss et al. [41], a three-arm trial that combined two vitamin D treatment groups (40,000 IU/week and 20,000 IU/week) into one vitamin D group, reported a significant increase in CRP after a 12-month intervention, compared to placebo. However, the calculated mean difference (Δ) between the combined vitamin D and placebo groups was not statistically significant for CRP (Δ 0.19 ± 1.25; p-value = 0.88). Inconsistent reporting was noted in the same study population by Jorde el al. 2010b [43], which reported no significant difference for cytokines between treatment groups, nor when combined vitamin D treatment groups were compared to placebo. In the remaining six trials, no significant mean differences (Δ) were found for CRP when vitamin D was compared to placebo.

**Table 4:** Mean differences for inflammatory biomarkers and glucose measures from randomized controlled trials.

Study (ref)	Treatment Arms†	No.	Vitamin D3 dose (IU); Frequency; Duration	Glucose measures Δ (SEM)		Insulin Measures Δ (SEM)			Inflammatory Markers Δ (SEM)				Adipokines Δ (SEM)	
				FPG	HbA1c	HOMA-IR	QUICKI	FIL	(hs-)CRP	TNF-α	IL-6	IL-10	Adiponectin	Leptin
				(mmol/L)	(%)			(pmol/L)	(mg/L)	pg/ml)			(mg/ml)	(ng/ml)
**Outcome: Inflammation and metabolic markers**														
Carrillo, 2012 [44]; Carrillo, 2013 [45]	I: VD+CA+SS+NS+PA; P: PL+CA+SS+NS+PA	I: 10; P: 13	4,000+100 (NS) Daily for 12 wks	-0.20(0.27)	-	0.40(0.69)	-	16.10 (18.21)	2.90(1.25)	-0.20 (0.51)	-0.20(0.27)	-	-	-
Nagpal, 2009 [49]	I: VDc; P: PLc (sachets)	I: 32; P: 33	120,000 Biweekly for 6 wks	-	-	-0.03(4.22)	0(0.00)	-	NR ⱡ	-	-	-	-	-
Wamberg, 2013 [47] ⱡⱡ	I: VD; P: PL	I: 22; P: 21	7,000 Daily for 26 wks	0.30(0.16)‡	-	-0.30(0.36)	-	-3.60 (5.69)	-3.0(1.71)	-	0.70(0.32)	-	0.40 (0.72)	5.40 (5.40)
		**NFG**		**NFG**		**NFG**			**NFG**		**NFG**			
Pittas, 2007 [46]	I: VD+CA; P: PL+CA	I: 108; P: 114	700 Daily for 3 yrs	0.03 (0.05)	-	NR ⱡ	-	-	-0.29(0.78)		-0.34(0.72)	-	-	-
		**IFG**		**IFG**		**IFG**			**IFG**		**IFG**			
		I: 45; P: 47		-0.32 (0.14)*	-	-0.86 (0.36)*	-	-	0.27(1.62)	-	0.30(1.05)	-	-	-
Zitterman, 2009 [48]	I: VDo + WRP; P: PLo + WRP	I: 82; P: 83	3,332 Daily for 1 yr	0.06(0.11)	0 (0.04)	-	-	-	0.02(0.09)	-0.54 (0.42)	-1.60(1.95)	-	-	-
Beilfuss, 2012 [41] § ⱡⱡ	I:DD/DP+CA+HD/PA; P: PP+CA+HD/PA	I: 220; P: 112	20,000 or 40,000 Weekly for 1 yr	-	-	-0.15(0.83)	0(0.003)	-	0.19(1.25)	0.03 (0.60)	-0.16(0.55)	-	-	-
Jorde, 2010a [42] §	DD: DD+CA+HD/PA; DP: DP+CA+HD/PA; PP: PP+CA+HD/PA	DD: 114; DP: 104; PP: 112		**DD**:-0.06 (0.06)	**DD:** 0 (0.03)	**DD**:-0.13 (0.59)	**DD:** 0 (0.003)	**DD**: -1.0 (12.69)	-	-	-	-	-	-
			DD: 40,000; DP: 20,000 Weekly for 1 yr	**DP:**0(0.06)	**DP:** 0.02 (0.03)	**DP:** -0.41 (0.36)	**DP:** 0 (0.003)	**DP:** -7.60 (7.19)	-	-	-	-	-	-
	**Subgroup analysis (IFG/IGT)** I: DD+DP; P: PP	I: 34; P: 31		-0.10(0.12)	0(0.06)	-0.60(0.92)	0(0.005)	-9.0(17.20)	-	-	-	-	-	-
**Outcome: Inflammation markers only**														
Jorde, 2010b [43] § ⱡⱡ	DD: DD+CA+HD/PA; DP: DP+CA+HD/PA; PP: PP+CA+HD/PA	DD: 104; DP: 98; PP: 105	DD: 40,000; DP: 20,000 Weekly for 1 yr	-	-	-	-	-	**DD:** 0.20(0.97)	-	-	**DD:** 0(6.50)	-	-
				-	-	-	-	-	**DP:** 0.20(1.40)	-	-	**DP:** 0(6.70)	-	-
Maki, 2011 [54]	I: MVMD; P: MVM	I: 31; P: 29	1,200 Daily for 8 weeks	-	-	-	-	-	-0.14(0.52)	-	-	-	-	-
**Outcome: Metabolic markers only**														
Davidson, 2013 [50]	I: VDs; P: PLs	I: 56; P: 53	Variable (88,865 mean) Weekly for 1 yr	-0.17 (NR)	-0.20 (NR)	-0.10 (NR)	-	-	-	-	-	-	-	-
Harris, 2012 [51]	I: VD+CA; P: PL+CA ◆	I: 43; P: 46	4,000 Daily for 12 weeks	0.02 (0.13)	0 (0.07)	0.38 (0.50)	-	12.09(9.33)	-	-	-	-	-	-
	I: VDCA+VDCP; P: VPCA + VPCP ◆◆	I: 46; P: 46		-0.18 (0.15)	-0.08 (0.04) §§	-	-	-	-	-	-	-	-	-
Mitri, 2011 [52]	**Subgroup Analysis 1**; I: VDCA; P: VPCP	I: 23; P: 24	2,000 Daily for 16 weeks	-0.31 (0.18)	-0.13 (0.06)*	-	-	-	-	-	-	-	-	-
	**Subgroup Analysis 2**; I: VD; P: PL + PL	I: 23; P: 23		-0.35 (0.19)	-0.10 (0.06)	-	-	-	-	-	-	-	-	-
Zhu, 2014 [53]	I: CA+VD+CR ◆; P: CR	I: 22; P: 21	125 Daily for 12 weeks	-0.09(0.10)	-	-	-	-8.46 (8.77)	-	-	-	-	-	-

**Δ** = Mean difference between groups

† **I** = Intervention arm; **P** = Placebo arm; **SEM** = Standard error of mean; **CA** = Calcium tablet (500 mg/day unless otherwise noted); **CR** = Calorie restriction (500 kcal/day); **DD** = 2 vitamin D tablets; **DP** = 1 vitamin D tablet and 1 placebo tablet; **HD/PA** = Written and oral information and recommendations on healthy diet and physical activity; **MVM** = Multivitamin and mineral; **MVMD** = Multivitamin and mineral with vitamin D; **NS** = Nutritional Shake (360 kcal, 8 g fat, 54 g carbohydrate, 20 g milk protein isolate, 100 IU vitamin D and 300 mg calcium); **PA** = Physical activity (progressive resistance training); **PL** = Placebo; **PLc** = Placebo sachets; **PLo** = Placebo Migliol oil; **PLs** = Placebo solution via oral syringes; **PP** = 2 placebo tablets; **SS** = Sunscreen; **VD** = Vitamin D tablet; **VDc** = Vitamin D sachets; **VDCA** = Vitamin D plus calcium; **VDCP** = Vitamin D plus calcium placebo; **VDo** = Vitamin D3 Vigantol oil; **VDs** = Vitamin D dissolved in median chain triglyceride via oral syringes; **VPCA** = Vitamin D placebo plus calcium; **VPCP** = Vitamin D placebo plus calcium placebo; **WRP** = Weight reduction program; **IFG** = Impaired fasting glucose (fasting plasma glucose 5.6 to 6.9 mmol/L); **NFG** = normal fasting glucose (fasting plasma glucose < 5.6 mmol/L); **IFG/IGT =** Impaired fasting glucose, and Impaired glucose tolerance, (fasting plasma glucose 6.0 to 6.9 mmol/L) and (2-hour plasma glucose 7.8 to 11.0 mmol/L)

**ⱡ NR** = Value not reported, but stated as a non-significant change

**§** Reference the same study population and protocol

**§§** Authors reported that excluding (n = 2) HbA1c outliers, the difference between vitamin D compared to no vitamin D became significant

ⱡⱡ Means not reported, median values and ranges provided

◆ = Calcium tablet (600 mg/day)

◆◆ = Calcium tablet (800mg/day)

**‡** Mean provided for FPG

* p < 0.05.

Further, within the vitamin D treatment groups, changes between pre-treatment and post-treatment within-group differences produced conflicting results. Three trials reported non-significant increases in CRP [44, 46, 54], while two trials observed decreases in CRP following vitamin D supplementation [47, 48]; only one resulting in a statistically significant change over-time [48]. Among the vitamin D intervention groups, CRP increased from baseline with sufficient baseline mean 25(OH)D concentrations and decreased with insufficient baseline mean 25(OH)D concentrations following vitamin D repletion. Similarly, Pittas et al. [46] stratified the analyses by baseline glucose tolerance status, and found that the mean difference (Δ) of CRP non-significantly increased between intervention groups among the IFG subgroup following vitamin D supplementation; however, decreased CRP means were observed among the NFG subgroup.

Three studies measured changes in TNF-α levels [41, 44, 48]. Vitamin D supplementation significantly lowered TNF-α concentrations over time in one study [48], but the mean difference was not statistically significant between the vitamin D and the placebo group (Δ -0.54 ± 0.42; p-value = 0.20).

Three of five trials measuring IL-6 reported reductions following vitamin D supplementation [41, 44, 48]; however, only one trial observed a significant reduction over time among those receiving vitamin D [48]. Further, vitamin D supplementation resulted in no statistically significant changes in levels of adipose-derived hormones, adiponectin and leptin [47]. Lastly, only one of the trials reported significant time-effects from baseline for inflammatory biomarkers, namely decreases in IL-6, TNF-α, and CRP levels were observed among the vitamin D with the lowest mean serum 25(OH)D (30.0 nmol/L ± 17.5 nmol/L) at baseline [48].

#### Glucose and insulin sensitivity measures

Of the eleven unique trials included in this review, FPG and HOMA-IR were the most commonly reported glucose and insulin sensitivity measures (10 of 11), followed by HbA1c and fasting insulin levels (Table 4). In two trials, vitamin D significantly decreased blood glucose measures (FPG, HbA1c) and insulin resistance (HOMA-IR), compared to placebo[46, 52]. Significant time-effects for glucose measures were observed in three trials following vitamin D supplementation, which resulted in either an attenuated increase or decrease in glycemic measures [48, 50, 53]. Pittas et al. [46] observed significant changes in FPG and HOMA-IR in a subsample of adults over 65 years of age with impaired fasting glucose (IFG). Among this subgroup, FPG significantly decreased when compared to placebo (Δ -0.32 **±** 0.14; p-value = 0.03); no significant differences were observed in the normal fasting glucose (NFG) subgroup. Over the 3-year period, FPG increased to a lesser degree among the vitamin D group as compared to the placebo group. Insulin resistance significantly decreased among the IFG subgroup (Δ -0.86 ± 0.36; p-value = 0.02). Specifically, HOMA-IR was effectively unchanged after 3-years in the calcium plus vitamin D treatment group, whereas HOMA-IR increased in the placebo group; no difference was observed between treatment groups in the NFG subgroup [46]. In a 16-week 2x2 factorial trial of adults with pre-diabetes, supplementation with vitamin D_3_ (2,000IU/day) and calcium carbonate (800 mg/day) resulted in non-significant attenuation of the rise in FPG and HbA1c levels compared to the control group [52]. In the subgroup analysis, however, HbA1c significantly decreased following treatment (Δ -0.13 ± 0.06; p-value = 0.04). In two other trials that also enrolled subjects with pre-diabetes [50, 51], HbA1c significantly decreased from baseline after 12-months of vitamin D supplementation [50], but there was no significant time-effects or absolute mean differences for glucose measures and insulin resistance following 12-weeks of supplementation [51].

Among non-diabetic participants, Zitterman et al. [48] reported a significantly greater decrease in FPG over time among the placebo group compared to the vitamin D group. In contrast, Carrillo el al. [45] observed a greater, albeit non-significant, reduction among the vitamin D group compared to placebo. Further, Zhu et al. [53] reported a significantly lower increase in FPG over time among those taking calcium plus vitamin D supplementation than among the control group, which had a higher percentage of obese young adults than overweight adults in the vitamin D treatment group compared to the control group, 22.2% and 10.5%, respectively. Finally, Wamberg et al. [47] reported non-significant diverging trends between treatment groups. Insulin resistance outcomes, as measured by HOMA-IR in six trials, were mixed. When compared to the control group, vitamin D supplementation resulted both in non-significant decreases [41, 42, 47, 49, 50] and increases [45, 51]. Furthermore, no significant findings were associated with QUICKI or FIL (n = 5), and no trials reported changes in EHC.

## Discussion

This systematic review assessed the effect of vitamin D supplementation (with or without calcium) on inflammatory markers, glucose and insulin sensitivity measures in overweight and obese adults among randomized controlled trials. Overall, the results of this systematic review did not find a clearly established benefit of vitamin D supplementation on inflammatory and glycemic markers among overweight and obese adults. Most of the reported trial outcomes were non-significant and included considerable bias. There was also substantial heterogeneity in trial design, participants and study outcomes. Of the 11 unique trial protocols included in this review, only two reported significant changes in markers of inflammation, i.e., within groups (endpoint vs. baseline); however, mean differences, as calculated in our analysis, were not statistically significant. Only two trials reported significant changes in glucose and insulin resistance following vitamin D_3_ supplementation when compared to placebo.

### Vitamin D, the inflammatory response and adiposity

The effect of vitamin D_3_ supplementation on CRP levels, the most commonly reported marker of inflammation, was mixed. Survey results from a nationally representative sample, National Health and Nutrition Examination Survey (NHANES), reported that vitamin D levels less than 21 ng/ml (< 52.42 nmol/L) had an inverse association with CRP levels among healthy adults in the United States; whereas, CRP levels greater or equal to 21 ng/ml had a positive association [62]. This cross-sectional finding aligns with the CRP results found in this systematic review. Furthermore, few trials measured changes in pro-inflammatory cytokine TNF-α. Vitamin D supplementation was shown to lower TNF-α levels in all trials from baseline albeit, the reduction was not always significant and the mean difference was never significant when compared to placebo. Similar inconsistencies have also been noted in other studies assessing the role of serum 25-hydroxyvitamin D on inflammatory cytokines [23, 24, 63].

### Vitamin D, glycaemia, insulin resistance and adiposity

The majority of trials included in this review reported glycemic outcomes (FPG, HbA1c). With the exception of one trial, mean FPG levels consistently increased less in the vitamin D group than placebo with sufficient baseline mean 25(OH)D concentrations and decreased with insufficient baseline mean 25(OH)D concentrations following vitamin D repletion, supporting the inverse association between vitamin D and glucose measure among overweight and/or obese individuals. This finding is consistent with other studies that show an inverse association between higher baseline 25(OH)D and glucose status [18, 21, 64]. Notably, the outlier trial was subject to differential loss to follow-up between treatment groups [47]; thereby, potentially impacting the clinical and statistical significance of these findings [60].

### Biological Mechanisms

Historically, the biological role of vitamin D was thought to be limited to calcium homeostasis and bone health [65]. However, there is considerable evidence suggesting that vitamin D plays several roles and can affect multiple organs and metabolic processes [66]. Vitamin D is one of few fat-soluble vitamins. Reduced vitamin D bioavailability is commonly observed in overweight and obese individuals, possibly the result of increased sequestration in adipose (fat) tissues [67, 68]. Vitamin D deficiency has also been strongly associated visceral adiposity [69]. Further, early works indicated that adipose derived-TNF-αwas not released into the circulation [70, 71]; however, recent evidence suggests that adiposity does translate into elevated serum TNF-α concentrations [71–73]. Visceral adiposity and associated cytokines secretion are posited to enter via the portal circulation in human and mouse models [15, 74–76]. Abdominal weight gain was prospectively shown to increase circulating TNF-a levels [77]. Similarly, overweight and obese women were observed to have higher serum TNF-α levels when compared to lean women [78]; also, subcutaneous and visceral adipocyte cell volume were shown to have a higher positive correlation with serum TNF-α levels in overweight/obese adults than lean controls [79]. Therefore, adipocyte hypertrophy in overweight and obese individuals may result in the increase synthesis and release of proinflammatory mediators (e.g. TNF-a, IL-6, etc.), thereby raising their circulating levels and promoting tissue inflammation [65, 73, 78–80]. Vitamin D deficiency, an indicator of low bioavailability of its active metabolite is thought to contribute to the pathogenesis of several obesity-related diseases through a variety of mechanisms. First, vitamin D has known immunoregulatory effects, suggesting it may modulate the inflammatory response in adipocytes [65]; For example, several studies have observed vitamin D (calcitriol) to inhibit production and expression of cytokines (e.g., IL-6, IL-8, and interferon gamma) [19, 65, 81–84]. Both vitamin D and its biologically active form have been shown to reduce lipopolysaccharide-induced TNF-α production, and further to regulate the activity of NFkB, which mediates the proinflammtory activities of TNF-α [19, 65, 73]. Supporting this, animal models have shown that calcitriol supplementation among weight induced mice suppress circulating proinflammatory cytokines TNF-α, IL-6, and CRP, which are posited to be mediated by vitamin D-related gene-level modifications [85]. Second, this inflammatory response further contributes to the pathogenesis of disease, particularly those related to glucose regulation such as metabolic syndrome and diabetes [18]. Specifically, there is evidence to suggest that the resulting elevation of proinflammatory cytokines may play a role in beta cell dysfunction, and further trigger beta cell apoptosis (i.e., cell death) [18]. Therefore, by directly modulating the production and effects of proinflammatory cytokines, vitamin D may potentially improve insulin sensitivity and promote beta cell survival [18, 65, 86, 87]. Indeed, several studies have demonstrated the ability of vitamin D supplementation to mediate changes in glucose metabolism through a combination of different biological processes, such as vitamin D receptor (VDR), feedback loops between parathyroid hormone (PTH) and calcium regulation, as well as altering the availability of biologically active metabolites [18, 19, 65, 86]. However, the role of vitamin D in the pathogenesis of obesity-related disease is complex and there has been mixed observational evidence supporting these biological mechanisms to date [19, 65, 88, 89]. Lastly, current advances relating to adipokines show adipose tissue cultures from mice treated with 1,25(OH)2D3 had increased stimulation of leptin in their tissue, resulting in increased serum leptin levels [90]; notably, serum leptin is reduced in mice without vitamin D receptors or the gene required to create active vitamin D [65]. Further, adiponectin in humans is observed to be positively correlated with serum vitamin D [65]. It is obvious from the literature that more research addressing the interaction between vitamin D and adipose tissue in humans is needed, as the implications of this association is yet to be fully elucidated [90, 91].

### Implications

Although the absolute effects of these trials are mixed, significant results from the small number of trials weakly support the idea that vitamin D may have a role in reducing the degree to which glycemia and insulin resistance measures increase over time in overweight and obese adults. The fact that these associations were only significant in a subpopulation with glycemia (pre-diabetes/IFG) suggest that this role may be more pronounced in individuals at increased risk of developing metabolic and obesity-related conditions. As previously supported in the literature, vitamin D may mitigate type-2 diabetes risk [92, 93]. These results demonstrate that potentially higher doses of vitamin D or doses over longer periods of time with a calcium co-intervention may be more beneficial towards inflammatory biomarkers and glucose outcomes, compared to lower doses over shorter periods of time.

One possible explanation for this finding lies in the expected biological dose-response of vitamin D supplementation, which varies according to different baseline serum 25(OH)D levels [94]. A wide range of vitamin D levels among subjects could potentially produce heterogeneous biological effects. Notably, half of the trials had a 25(OH)D levels less than 50 nmol/L and among trials reporting significant glucose or inflammatory biomarkers, only one trial had mean baseline 25(OH)D less than 50 nmol/L in both intervention groups. Moreover, latitude impacts UV exposure, and consequently, those, in higher latitude areas have lower serum 25(OH)D concentrations during fall and winter months [95]. Two of the trials that reported significant inflammatory marker changes over time occurred in Norway and Germany; thereby, strengthening the argument that lower 25(OH)D baseline concentrations potentially play an important role in the expected response to vitamin D supplementation. Noteworthy, participants who dropped out from one of the trials were significantly younger, more commonly smokers, with higher BMI (p-values < 0.05) and lower serum 25(OH)D levels (p-value < 0.001) [41–43]. These characteristics have been shown to influence inflammation and glucose measures, potentially biasing the results toward the null. Many of the trials had unclear or high risks of bias; thereby, potentially influencing the validity of the reported results. Trials with non-significant results were possibly underpowered (end of study sample size < 100) to detect a differences [94]. As well, a lack of associations for a specific risk factor could be related to a dilution effect; i.e., when overweight or obese individuals are grouped together, the “one size does not fit all” concept occurs [96]. Therefore, there may be merit in individually assessing the role of vitamin D supplementation on outcomes measures within specific weight subgroups, as stratified by appropriate age-specific subgroups.

Recent attention has been directed towards the divergent results of vitamin D between prospective studies and randomized trials; indeed, this systematic review also shows inconsistencies for markers of inflammation (i.e. CRP, IL-6), glucose measures and insulin resistance (i.e. HbA1c, HOMA-IR). The association between 25(OH)D concentrations and various health outcomes as reported among systematic reviews and meta-analyses from observational studies and randomized controlled trials provide “suggestive” evidence of an association between vitamin D and various obesity-related outcomes (i.e. cardiovascular disease, metabolic syndrome and type-2 diabetes) [97]. One possible explanation for this difference is that clinical disease actually reduces vitamin D concentrations, producing insufficiency, rather than causing ill health [97].

### Limitations

This systematic review is strengthened by the inclusion of randomized controlled trials in conducted in recent years, as well as the inclusion of numerous inflammatory and glycemic outcomes. However, several limitations exist at the study, outcome and review level. The trials included in this review were heterogonous according to the types of outcome measures reported, vitamin D dosages, duration of supplementation, and quality. Considering the diverse treatments and comparators in each of the trials, we were not able to perform a meta-analysis. This review was also limited by the small number of eligible studies, with many of the included trials not originally designed for the primary outcomes intended for this review. The primary objective of many of these trials was to investigate body weight, muscle function and skeletal outcomes, as opposed to specifically testing the association of vitamin D and the measures reported here. The effect of vitamin D supplementation on cytokines was predominately among Caucasian populations; no trials measured inflammatory markers within ethnic subgroups.

Several studies noted high dropout rates, which may affect the internal validity of these findings. While studies with small sample sizes and non-significant results are less likely to be published [98], this was not evident in this systematic review. As previously discussed, many of the trials were classified as “high risk” or “unclear risk” of bias; this affects the validly of the findings and, subsequently, the recommendations that are suggested from the results [58]. Additionally, industry sponsored trials have been shown to be prone to bias [99]; half of the trials included in this review were reported as being sponsored by industry, including two which reported significant results.

The methodological rigor for this systematic review is supported by the following: i) protocol development and consensus prior to initiating the literature search; ii) clear *a priori* inclusion and exclusion criteria; iii) preferred reporting of PRISMA for systematic reviews and meta-analyses guidelines were followed [30]; and iv) collaborations with a professional librarian made it possible for a comprehensive and extensive search on electronic databases, limiting search bias [100]. However, there is always the potential that not all RCTs among obese/overweight relating to inflammatory biomarkers and glycemic measures were identified, increasing the likelihood of indexing bias [100]. Furthermore, we identified multiple publications generated from a single trial, suggesting potential publication biases in the literature. This systematic review did contain two trial protocols that published multiple publications, but the trials did not follow any significant pattern of increasing positive results [100].

### Recommendations

In order to further elucidate the role of vitamin D supplementation on inflammatory and glycemic outcomes among RCTs, future randomized controlled trials should focus on improving the quality of the study design; we identified very few studies classified as having low risk of bias. Secondly, trialists should consider designing trials specific for vitamin D assessment, as most of the trials reviewed had two or more trial interventions (i.e. weight loss, resistance or exercise training interventions, with oral vitamin D supplementation). Trials had relatively small sample size with predominately white participants and variable vitamin D dosages and duration. We recommend longer intervention periods of clinically safe higher doses, attention to different ethnic groups, and specific, clear aims with a sole focus on vitamin D intervention and primary inflammatory outcomes. Third, we recommend trialists consider analysis of vitamin D supplementation in trials with obese or overweight subjects, consider subdividing overweight and obese patients into smaller categories, with additional sex and age-specific subgroups. Lastly, we recommend further considerations to baseline vitamin D concentrations at the time of enrollment, which are necessary to capture varying categories of vitamin D status.

## Conclusion

We identified eleven unique trials assessing the role of vitamin D supplementation on various inflammatory and glycemic outcomes among overweight and obese adults. Overall, the results of this systematic review do not clearly establish a benefit of vitamin D supplementation on inflammatory and glycemic markers in this population. However, there is some indication that baseline serum vitamin D influences the effect of vitamin D repletion on inflammatory markers. Additional, high-quality studies specifically designed to assess the role of vitamin D on relevant inflammatory and glycemic measures are required to ascertain whether vitamin D supplementation has any clinical benefit among overweight and obese adults.

## Supporting Information

S1 File Search Strategy. (PDF)

S2 File PRISMA Checklist. (PDF)
