# Supplementary material for: Effect of Vitamin D3 Supplementation on Inflammatory Markers and Glycemic Measures among Overweight or Obese Adults: A Systematic Review of Randomized Controlled Trials
Source: PLoS One. 2016 Apr 26;11(4):e0154215. doi: 10.1371/journal.pone.0154215 (PMC4846157; doi:10.1371/journal.pone.0154215)
Supplement: S1 File — (PDF) [file pone.0154215.s001.pdf]

S1 File. Search Strategy

Database: Ovid MEDLINE(R) <1946 to October Week 40 2013>, Ovid MEDLINE(R) In-Process & Other Non-Indexed Citations <October 01, 2013>
Search Strategy:

1 exp Obesity/ [overweight / obesity terms] (141564)
2 Overweight/ (12056)
3 exp "Body Weights and Measures"/ (446842)
4 adiposity.tw. (14375)
5 (body adj fat$).tw. (22788)
6 (body adj weight).tw. (148287)
7 "body mass index".tw. (100979)
8 BMI.tw. (74228)
9 (excess$ adj weight$).tw. (4003)
10 obes$.tw. (176826)
11 overweight$.tw. (38317)
12 over-weight$.tw. (347)
13 (skinfold adj thickness$).tw. (3681)
14 (skin-fold adj thickness$).tw. (682)
15 (waist adj circumference$).tw. (14292)
16 "weight to height ratio$".tw. (222)
17 "waist to hip circumference$".tw. (1543)
18 (waist-hip adj ratio$).tw. (2647)
19 or/1-18 (656489)
20 exp Vitamin D/ [vitamin D terms] (44441)
21 exp Cholecalciferol/ (23535)
22 exp Ergocalciferols/ (3364)
23 exp Dihydrotachysterol/ (633)
24 exp 25-hydroxyvitamin D 2/ (624)
25 exp Hydroxycholecalciferols/ (19391)
26 ("25" adj hydroxy$).tw. (11365)
27 alphacalcidol$.tw. (138)
28 alfacalcidol$.tw. (692)
29 cholecalciferol$.tw. (1559)
30 colecalciferol$.tw. (31)
31 calcifediol$.tw. (59)
32 calcitriol$.tw. (3877)
33 calciol$.tw. (20)
34 calcamine$.tw. (0)
35 dihydrotachyster$.tw. (340)
36 ergocalciferol$.tw. (417)
37 ercalcidiol$.tw. (2)
38 (hydroxyvitamin$ adj D).tw. (6735)
39 (hydroxyvitamin$ adj D2).tw. (158)
40 (hydroxyvitamin$ adj D3).tw. (2075)
41 hydroxycholecalciferol$.tw. (1294)
42 tachystin$.tw. (2)
43 (vitamin adj D).tw. (38625)
44 (vitamin adj D2).tw. (1029)
45 (vitamin adj D3).tw. (6938)
46 or/20-45 (62574)
47 (clinical adj trial).mp. [RCT validated filter - highly sensitive] (603048)
48 clinical trial.pt. (504366)
49 random$.mp. (921575)
50 tu.xs. (3573461)
51 or/47-50 (4232019)
52 19 and 46 and 51 (2490)
53 exp Animals/ not (exp Animals/ and Humans/) [removing animal studies] (4050087)
54 52 not 53 (1948)

In collaboration with Laure Perrierr (librarian) at Li Ka Shing Knowledge Institute. St. Michael's Hospital, Toronto, Ontario.

Database: EMBASE <1947 to October Week 40 2013>, Ovid MEDLINE(R) In-Process & Other Non-Indexed Citations <October 01, 2013>
Search Strategy:

1 exp Obesity/ (286121)
2 Weight Gain/ (64362)
3 Waist Hip Ratio/ (6424)
4 Weight Height Ratio/ (21)
5 Waist Circumference/ (20632)
6 Skinfold Thickness/ (8551)
7 adiposity.tw. (17414)
8 (body adj fat$).tw. (27835)
9 (body adj weight).tw. (194037)
10 "body mass index".tw. (124266)
11 BMI.tw. (120571)
12 (excess$ adj weight$).tw. (5807)
13 obes$.tw. (234206)
14 overweight$.tw. (50921)
15 over-weight$.tw. (692)
16 (skinfold adj thickness$).tw. (4391)
17 (skin-fold adj thickness$).tw. (953)
18 (waist adj circumference$).tw. (19955)
19 "weight to height ratio$".tw. (289)
20 "waist to hip circumference$".tw. (2029)
21 (waist-hip adj ratio$).tw. (3483)
22 or/1-21 (661060)
23 exp Vitamin D/ (92222)
24 ("25" adj hydroxy$).tw. (13908)
25 alphacalcidol$.tw. (122)
26 alfacalcidol$.tw. (622)
27 cholecalciferol$.tw. (2112)
28 colecalciferol$.tw. (102)
29 calcifediol$.tw. (95)
30 calcitriol$.tw. (4851)
31 calciol$.tw. (25)
32 calcamine$.tw. (28)
33 dihydrotachyster$.tw. (690)
34 ergocalciferol$.tw. (644)
35 ercalcidiol$.tw. (2)
36 (hydroxyvitamin$ adj D).tw. (7501)
37 (hydroxyvitamin$ adj D2).tw. (232)
38 (hydroxyvitamin$ adj D3).tw. (2777)
39 hydroxycholecalciferol$.tw. (1583)
40 tachystin$.tw. (24)
41 (vitamin adj D).tw. (51306)
42 (vitamin adj D2).tw. (2013)
43 (vitamin adj D3).tw. (10754)
44 or/23-43 (105704)
45 random$.tw. (872727)
46 placebo$.mp. (329231)
47 double-blind$.tw. (150845)
48 or/45-47 (1088853)
49 22 and 44 and 48 (1305)
50 Nonhuman/ (4146485)
51 49 not 50 (1149)

In collaboration with Laure Perrierr (librarian) at Li Ka Shing Knowledge Institute. St. Michael's Hospital, Toronto, Ontario.

Database: Cochrane Central Register of Controlled Trials (CENTRAL) CENTRAL <1inception to October Week 41 2013>, CENTRAL In-Process & Other Non-Indexed Citations <October 07, 2013> Search Strategy:

Cochrane

exp Obesity
Overweight/
exp "Body Weights and Measures"/

adiposity or "body fat*" or "body weight" or "body mass index" or BMI or "excess* weight*" or obes* or overweight* or over-weight* or "skinfold thickness*" or "skin-fold thickness*" or " waist circumference*" or "weight to height ratio*" or "waist to hip circumference*" or "waist-hip ratio*"

AND

exp Vitamin D/
exp Cholecalciferol/
exp Ergocalciferols/
exp Dihydrotachysterol/
exp 25-hydroxyvitamin D 2/
exp Hydroxycholecalciferols/

"25 hydroxy*" or alphacalcidol* or alfacalcidol* or cholecalciferol* or colecalciferol* or calcifediol* or calcitriol* or calciol* or calcamine* or dihydrotachyster* or ergocalciferol* or ercalcidiol* or "hydroxyvitamin* D" or "hydroxyvitamin* D2" or "hydroxyvitamin* D3" or hydroxycholecalciferol* or tachystin* or " vitamin D" or " vitamin D2" or "vitamin D3"

In collaboration with Laure Perrierr (librarian) at Li Ka Shing Knowledge Institute. St. Michael's Hospital, Toronto, Ontario.
